# Prone-transpsoas as single-position, circumferential access to the lumbar spine: A brief survey of index cases

**DOI:** 10.1016/j.xnsj.2021.100053

**Published:** 2021-02-19

**Authors:** Lauren E. Stone, Arvin Raj Wali, David R. Santiago-Dieppa, William R. Taylor

**Affiliations:** Department of Neurosurgery, University of California, 9300 Campus Point Drive, Mail Code 7893 La Jolla, CA, USA

**Keywords:** Single position surgery, Minimally invasive surgery, Lumbar spine, Spinal deformity, PTP, Prone transpsoas, T2DM, Type two diabetes mellitus, HTN, hypertension, HLD, hyperlipidemia, PI, pelvic incidence, LL, lumbar lordosis, ACR, anterior column release, PT, pelvic tilt, SVA, sagittal vertical axis, ALIF, anterior lumbar interbody fusion, EBL, estimated blood loss

## Abstract

**Background case description:**

Prone transposoas (PTP) is a novel approach to the lateral lumbar interbody fusion that harnesses the advantages of minimally invasive surgery with circumferential access to the lumbar spine in a single position. We present the peri-operative course of four index cases of patients having undergone PTP at a single institution.

**Outcome:**

Pre and post-operative spinal imaging with alignment parameters, operative approach, and patient outcome are reviewed for each index case.

**Conclusion:**

As advances in neuromonitoring and minimally invasive technology continue to evolve, new lumbar interbody fusion approaches are becoming operatively feasible.

## 1 Introduction

Interbody fusion is an operative staple to mitigate lumbar instability. Multiple approaches to the interbody space are currently practiced with new advances in minimally invasive surgery further introducing viable access corridors. The prone transpsoas (PTP) approach has emerged as part of this evolution, harnessing a minimally invasive approach to the interbody space while simultaneously providing circumferential access to the lumbar spine in a single, familiar position. Our early experience with PTP has demonstrated its broad application for both simple and complex spinal pathology. In this paper, we begin with a brief overview of the PTP approach followed by four demonstrative cases of PTP, detailing the multi-faceted access capable with this approach without compromising patient outcome or morbidity.

## 2 Prone transpsoas operative approach

A detailed discussion of the PTP approach as been published elsewhere [1]. The patient is positioned prone on a standard Jackson table. Needle/surface electrodes are placed for saphenous nerve SSEP monitoring and triggered EMG. The patient is draped for access to both the lateral and posterior spine. Fluoroscopy is used to identify the disc space in question. Retroperitoneal access is obtained avia finger dissection after the fascia is sharped incised. The initial dilator is placed in this retroperitoneal space to the psoas surface and sequentially dilated w/triggered EMG and fluoroscopy evaluation. The orthogonality of the dilating is critical for ensuring appropriate access to the interbody space.

A k-wire is then inserted into the disc space and a specialized access system (ATEC Spine, Carlsbad, CA) is advanced and locked into position on the table. Under the guidance of direct illumination, the surgeon may then prepare the disc space in the standard fashion with subsequent implant sizing and placement. Ongoing saphenous SSEP monitoring gauge retraction fatigue on the lumbar plexus. Fluoroscopy is used to confirm the appropriate apposition and alignment of the hardware.

Throughout this process, a co-surgeon can access the posterior spine for placement of either percutaneous hardware or other minimally invasive or open interventions as dictated by the pathology in question.

The lateral and posterior incisions are then closed in a standard fashion.

## 3 Case series

### 3.1 Case one

**Fig. 1:**
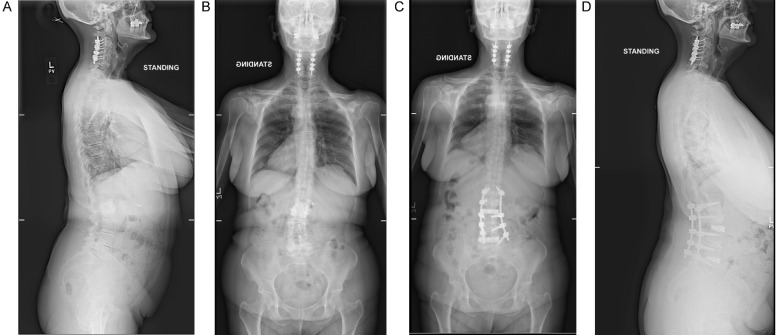
A: pre-operative AP full cassette scoliosis film demonstrating coronal imbalance. B: pre-operative lateral full cassette scoliosis film demonstrating normal sagittal balance. C: post-operative AP full cassette scoliosis film demonstrating correction of coronal imbalance and interval placement of L1-L5 pedicle screws. D: post-operative AP full cassette scoliosis film demonstrating maintenance of sagittal balance and lumbar lordosis with placement of interbody cages.

The patient is a 67-year-old female with type two diabetes mellitus (T2DM), hypertension (HTN), osteopenia (Z score -.7) with progressive left greater than right lower extremity radiculopathy extending to her feet. The patient was unable to walk for more than 5 min before experiencing aching pain in her legs, refractory to medications and physical therapy. Pre-operative imaging revealed adult degenerative scoliosis at L5-S1 with grade 1 spondylolisthesis at L5-S1, diffuse degenerative disc disease, and moderate lumbar stenosis. She had no fractional curve, and normal discs at T12-L1 and L5-S1. Scoliosis imaging revealed a coronal Cobb angle of 24°, pelvic incidence (PI) I 59°, lumbar lordosis (LL) 30° (PI-LL mismatch 29°), pelvic tilt (PT) 22°, and sagittal vertical axis (SVA) of 7 cm. Segmental lordosis at L3-4 was 2.8°, L4-5 5° (Fig. 1a and b).

Sher underwent a PTP approach for an L1-5 lateral interbody fusion interbody with simultaneous percutaneous pedicle screws at all levels with aims to (1) indirectly decompress her modest lumbar stenosis and neural foramen to reduce radiculopathy and (2) correct the coronal imbalance. A maximally lordotic, 10° cage was placed L3-4 to augment the lordosis obtained by prone positioning. A flat cage was used at L1-2 for the purpose of distraction. There were no intraoperative complications. Estimated blood loss (EBL) was 200 cc's. Post-operatively, the patient developed subtle bilateral thigh weakness, which resolved after 48 h. She was discharged on post-operative day 5 due to a *clostridium difficile* infection, presumably due to peri-operative antibiotic. Subsequent scoliosis films revealed a Cobb angle of 0°, PI 61°, LL 57°, PT 22°, SVA 0 cm. Segmental lordosis at L3-4 was 14.2°, L4-5 17° (Fig. 1c and d).

### 3.2 Case two

**Fig. 2:**
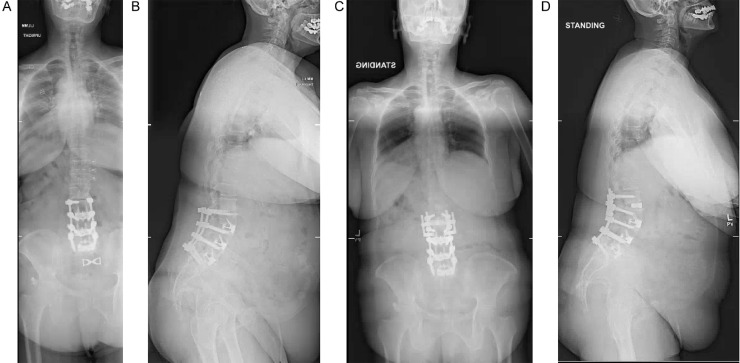
A: pre-operative AP full cassette scoliosis film demonstrating prior L1-3 ALIF with L1-3 posterior fusion. B: pre-operative lateral full cassette scoliosis film demonstrating PI-LL mistmatch and lumbar kyphosis. C: post-operative AP full cassette scoliosis film demonstrating placement of 10 degree cage and extension of hardware at L2-3. D: post-operative AP full cassette scoliosis film demonstrating reduction in PI-LL mismatch and mild correction of SVA.

The patient is a 62-year-old female T2DM, HTN, prior L1-3 anterior lumbar interbody fusion (ALIF) with L1-3 pedicle screws, presenting with progressive low back pain and right greater than left thigh radicular pain. She was unable to walk more than 100 yards or stand for more than two minutes without debilitating pain. Physical exam revealed bilateral 4/5 strength at hip flexion, dorsiflexion, and big toe flexion. Scoliosis films revealed lumbar kyphosis with PT 30°, PI of 66°, LL off 33° (PI-LL mismatch = 22°) and SVA of 10cm. Segmental lordosis at L2-3 was 7° (Fig. 2a and b).

The patient underwent an L2-3 PTP with anterior column release with placement of lordotic, porous titanium cages, replacement of posterior hardware and extension of previous laminectomies to L2-3 with facet releases at these same levels. The lateral and prone interventions were performed in parallel, therefore augmenting lordotic reduction on the table and allowing placement of a 10° cage at L2-3. EBL was 200 cc's. The patient was discharged post-operative day 2 without complications. On post-operative scoliosis films, her post-operative SVA was 6cm, PI 57°, LL 53° (PI-LL mismatch of 4), and PT 13°. Segmental lordosis across L2-3 was 22° (Fig. 2c and d).

### 3.3 Case three

**Fig. 3:**
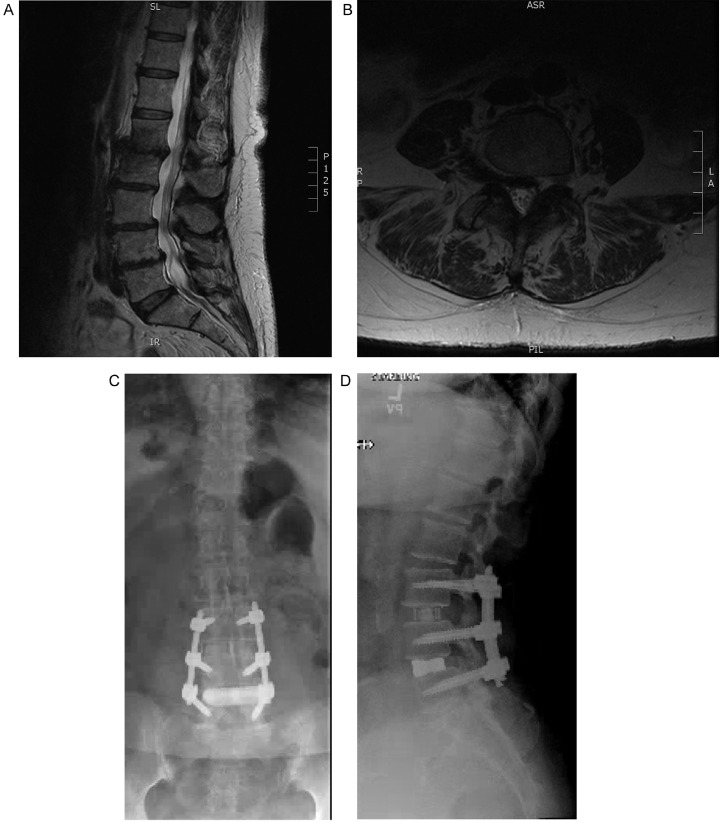
A. pre-operative sagittal T2 MRI of the lumbosacral spine with an L4-5 spondylolisthesis and degenerative disc disease at multiple levels in the lumbar spine, including grade four facet joint deterioration at L4-5 with locked facets. B: pre-operative axial T2 MRI of the lumbosacral spine with an L4-5 spondylolisthesis and degenerative disc disease at multiple levels in the lumbar spine, including grade four facet joint deterioration at L4-5 with locked facets. C: post-operative AP lumbosacral X-ray demonstrating interval placement of interbody cage and posterior instrumented fixation. D: post-operative lateral lumbosacral X-ray demonstrating multilevel interbody cage placement and pedicle screw fixation with maintained alignment of the lumbar spine.

The patient is a 71-year-old female with a history of T2DM, HTN, hyperlipidemia (HLD), with progressive, symmetric bilateral lower extremity pain. She had undergone lumbar epidural steroid injections, physical therapy, and medical management of the pain but continued to experience worsening symptoms. Physical exam revealed paresthesia's in the bilateral L4-S1 distribution and a positive bilateral straight leg raise. MRI revealed an L4-5 spondylolisthesis and degenerative disc disease at multiple levels in the lumbar spine, including grade four facet joint deterioration at L4-5 with locked facets (Fig. 3a and b).

Operative intervention consisted of lateral interbody access to L3-5 for correction of spondylolisthesis and lumbar lordosis, with L3-5 laminectomies for direct decompression given her stenosis and locked facets. A 10° cage was placed at L3-4 for the purposes of augmenting lordosis achieved with prone positioning. Bilateral percutaneous screws at L3-5 were placed through posterior fascial incisions. The above anterior and posterior column access were achieved simultaneously with two working surgeons. Intraoperative SSEPs showed decreases amplitude during psoas retraction, which spontaneously resolved with docking removal. EBL was 100 cc's. The patient was discharged post-operative day 2 without complications and resolution of her paresthesia. Post-operative films revealed successfully placed, wide footprint interbody spacers with reduction of her multi-level spondylolisthesis (Fig. 3c and d).

### 3.4 Case four

**Fig. 4:**
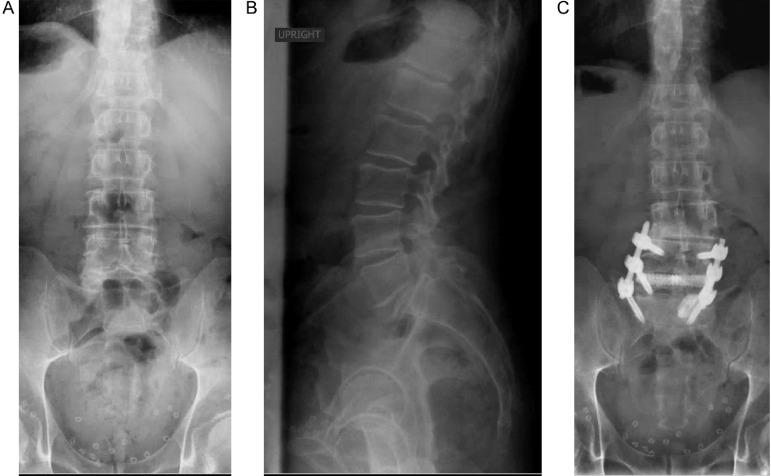
A: pre-operative AP x-ray of the lumbosacral spine demonstrating multilevel spondylolisthesis and degenerative disease. B: pre-operative lateral x-ray of the lumbosacral spine demonstrating multilevel spondylolisthesis and bilateral pars defect at L5-S1. C: post-operative AP x-ray of thoracolumbar spine film demonstrating placement of TLIF cage and pedicle screws. D: post-operative lateral x-ray of thoracolumbar spine with interval placement of TLIF cage and modest correction of spondylolisthesis.

The patient is a 72-year-old male with history of HTN with a decade of progressive low back pain . Physical exam revealed right greater than left radicular pain in the L4-S1 distributions. Imaging revealed a bilateral pars defect at L5-S1 with associated spondylolisthesis, spondylolisthesis at L4-5, and degenerative disc disease at L3-L5 with multilevel foraminal stenosis (Fig. 4a and b).

Operative intervention was planned to avoid fusion at L3-4 with focus placed on decompressing the symptomatic levels on the right L3-S1. The patient underwent a PTP lateral interbody fusion at L4-5, with minimally invasive foraminotomies and laminotomies at these levels and L5-S1 transformational lumbar interbody fusion. EBL was 200 cc's. He was discharged post-operative day two with resolution of his pain (Fig. 4c and d).

## 4 Discussion

Prone transpsoas is a novel approach to circumferentially address complex lumbar pathology in a familiar, accessible, single position procedure. The cases in this report are a sample of a single institution's early experience with PTP, demonstrating promising outcomes in a wide variety of lumbar pathology, including locked facets, degenerative disc disease, adult degenerative scoliosis, and fixed kyphosis.

**Fig. 5:**
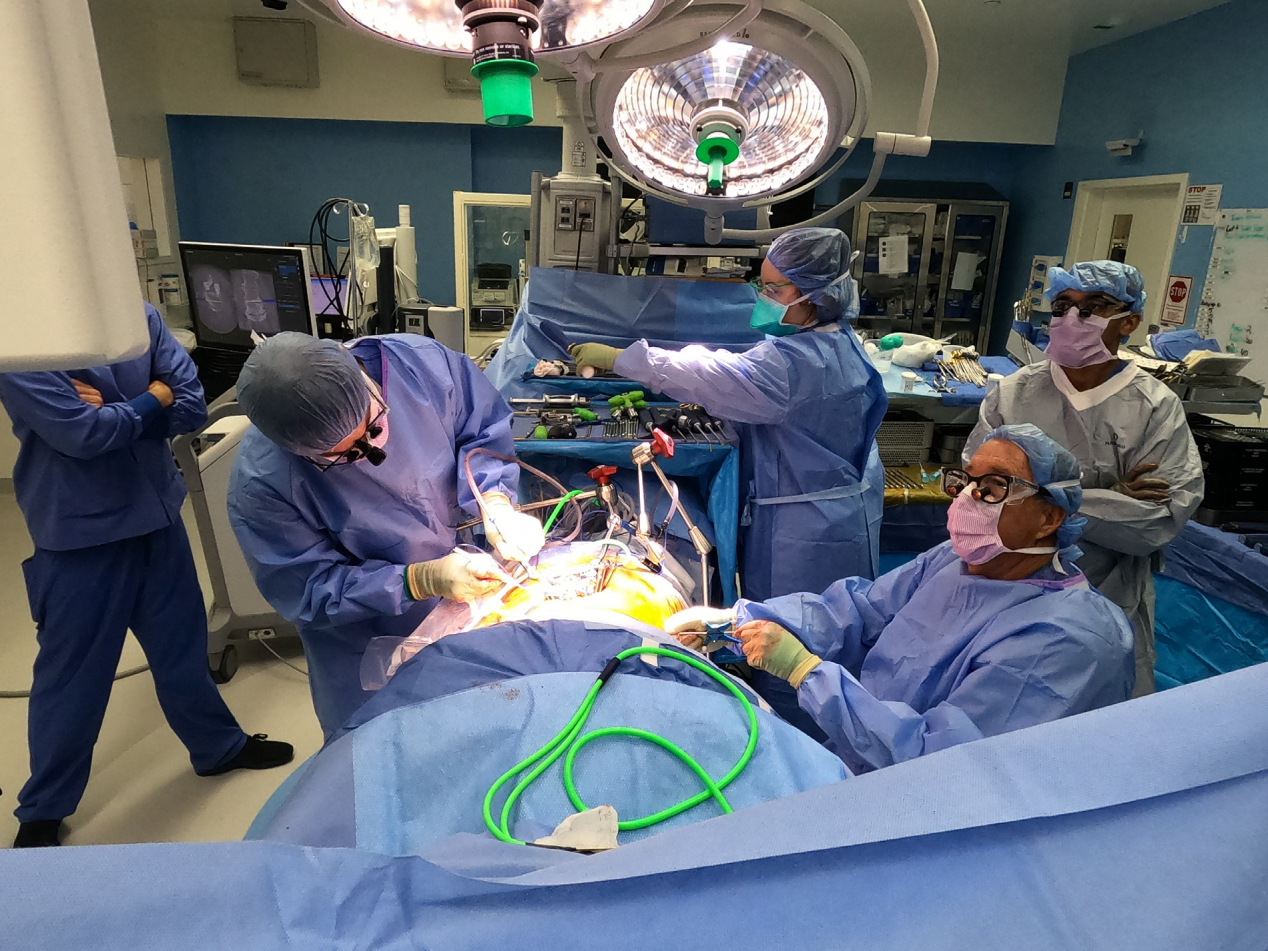
Demonstration of room set-up for simultaneous PTP access to lumbar interbody and posterior spine.

The advantages of PTP are multifold, including (1) familiar positioning, (2) the ability to augment lordotic reduction via prone-induced gravity, (3) minimally invasive access with an associated reduced risk profile compared to open interbody fusion, and (4) wide disc space access for maximal cage footprint and lordotic angling. PTP uniquely harnesses these strengths in parallel, thus synergizing the releases, gravity, and lumbar cage implantation to grant greater correction of lumbar lordosis than could be achieved otherwise. While an in depth overview of PTP technique and merits are published elsewhere this paper offers our early experiences of PTP, suggesting substantial possibilities for lordotic correction, akin to if not greater than those reported for other open approaches to the lumbar spine [1,2]. (Fig. 5).

**Fig. 6:**
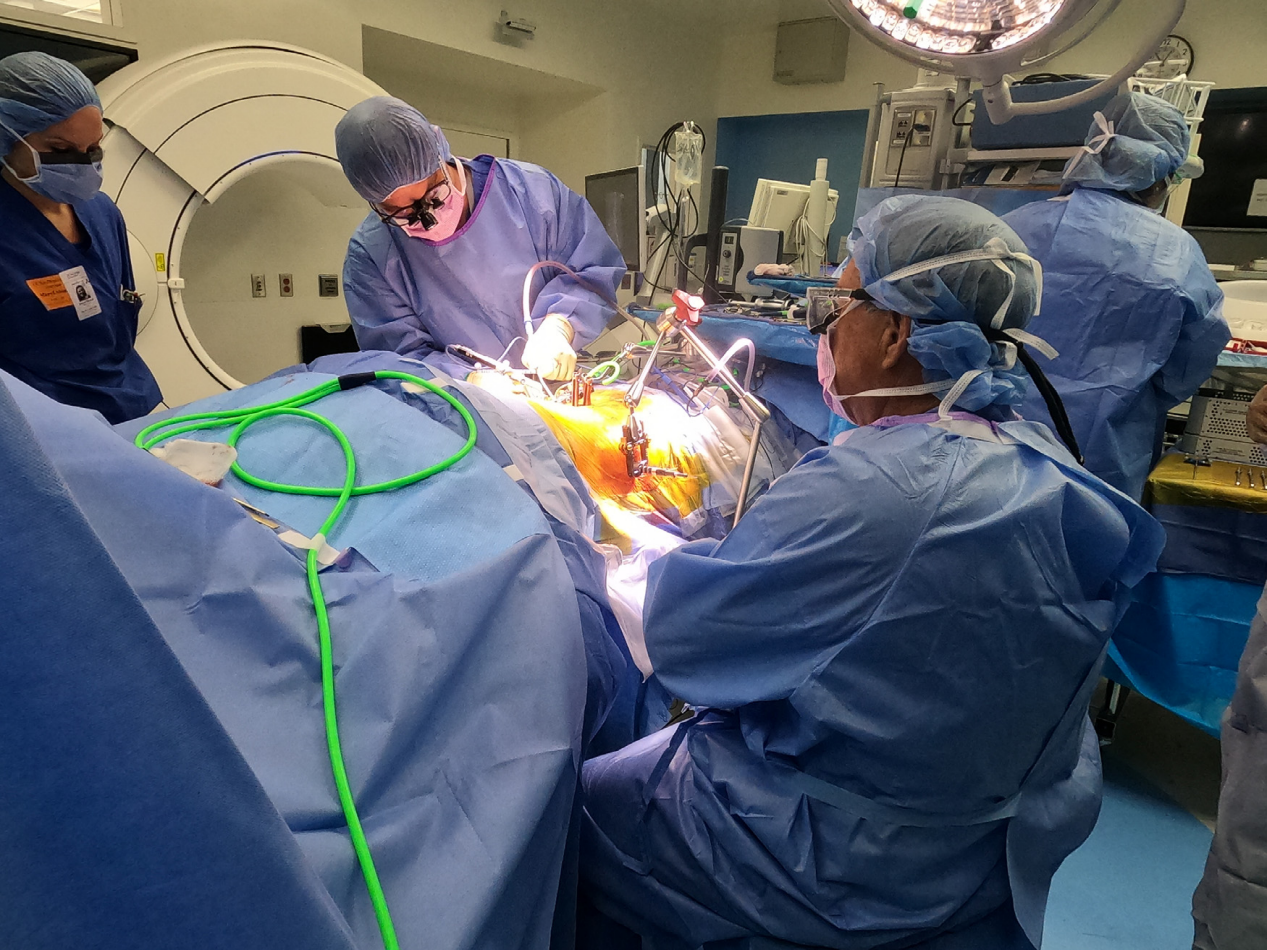
Surgeons have the choice to sit or stand during PTP. Ample space is available for staff maneuverability in a simplified set-up.

The benefits sourced from single position surgery lie in the ability to address circumferential pathology without timely repositioning. Several operative corridors have been proposed prior to PTP, including approaches in the decubitus position (OLIF, XLIF, LLIF). However, PTP uniquely starts from a familiar, prone, position, lessening the learning curve for surgeons and operative staff. Our experience further suggests that this gain in familiarity does not come at a cost of access. Case one articulates this well, demonstrating the synergism of indirect and direct decompression to open the neural foramen and fully decompress the thecal sac [3]. Fig. 6).

Our series also demonstrates that PTP can provide modest deformity correction. As a variant of lateral interbody fusion, the transpsoas portion of PTP achieves coronal correction, especially when coupled with bilateral pedicle screws [4]. Sagittal correction is modest, though augmentable with releases [5]. Case one of this series provides an example of effective deformity correction with multi-level interbody fusion, without releases. Case two provides an example of both sagittal and coronal deformity, corrected with a combination of releases, positioning and lordotic cages. Taken together, these two cases demonstrate PTP's ability to address spinal alignment effectively, without requiring repositioning or complex positioning.

Finally, PTP is classified as a minimally invasive surgery carrying the expected, lower risk MIS outcome profile. All estimate blood losses is this series were less that 200 cc's [6]. No patient experienced a retroperitoneal hematoma, although this, as similar to other lateral procedures, is a potential complication if care is not taken to note the segmental arteries lateral to the vertebral body. Length of stay was also minimal, with the exception of case two due to a clostridium difficile infection. As we are one of the first centers to practice PTP, the above cases are fresh in our repository and therefore require long-term follow up to further delineate patient outcomes.

## 5 Conclusion

Our experience with the prone transpsoas approach demonstrates the broad applications of simultaneous lateral and posterior access while maintaining a minimally invasive side effect profile. Additional long-term term follow-up will better delineate patient outcomes, which at this time appear promising.

## Conflicts of Interest

Dr. Taylor is a consultant of a company involved in the manufacture of the devices described as facilitating the surgical technique herein and receives royalties from the same company.

## Financial and Material Support

The project was completed without financial support.

## Informed patient consent

The authors declare that informed patient consent was taken from all the patients.

## Appendix Supplementary materials

Image, application 1

Image, application 2
