# Supplementary material for: Prone-transpsoas as single-position, circumferential access to the lumbar spine: A brief survey of index cases
Source: N Am Spine Soc J. 2021 Feb 19;6:100053. doi: 10.1016/j.xnsj.2021.100053 (PMC8820054; doi:10.1016/j.xnsj.2021.100053)
Supplement: Supplementary file 1 [file mmc1.pdf]

NASSJ

The Open Access Journal of the North American Spine Society

FDA Approval Status of Devices/Drugs

Article Title: Prone-Transpsoas as Single-Position, Circumferential Access to the Lumbar Spine: A Brief Su

Manuscript reference #: PENDING

FDA Approval Status

If a device or drug requiring FDA approval is identified as an important component of your article, you must indicate the FDA status for use as it will be discussed. Please list the name of the device(s) and drug(s) requiring FDA approval and check the appropriate status for use as it is discussed in the article.

[x] My manuscript does not discuss any drugs or devices requiring FDA approval.

1. Device/Drug: N/A

◯ Not Applicable ◯ Not approved for this indication ◯ Approved ◯ Investigational

2. Device/Drug:

◯ Not Applicable ◯ Not approved for this indication ◯ Approved ◯ Investigational

3. Device/Drug:

◯ Not Applicable ◯ Not approved for this indication ◯ Approved ◯ Investigational

4. Device/Drug:

◯ Not Applicable ◯ Not approved for this indication ◯ Approved ◯ Investigational

5. Device/Drug:

◯ Not Applicable ◯ Not approved for this indication ◯ Approved ◯ Investigational

Corresponding Author Name: Date:
